# Supplementary material for: Pharmacophore modeling and QSAR analysis of anti-HBV flavonols
Source: PLoS One. 2025 Jan 13;20(1):e0316765. doi: 10.1371/journal.pone.0316765 (PMC11730388; doi:10.1371/journal.pone.0316765)
Supplement: Attachment — Submitted filename: Response-to-reviewers.docx [file pone.0316765.s003.docx]

**September 19, 2024**

**PONE-D-24-19041**

**Title:** Ligand-based pharmacophore modeling, virtual screening, and 2D quantitative structure-activity relationship performance on anti-Hepatitis B virus flavonols

**Journal:** PLOS ONE

**Response to Reviewers**

Dear Dr. Nafees Ahemad,

We thank you for the opportunity to revise our manuscript and the valuable feedback during the review process. We have carefully considered all the comments and have made the necessary revisions to improve the manuscript. Below, we provide a detailed response to each reviewer's comment.

Journal Requirements:

1. Please ensure that your manuscript meets PLOS ONE's style requirements, including those for file naming.

**Action taken:** We have revised our manuscript to comply with PLOS ONE's style requirements, including appropriate file naming conventions.

2. In the online submission form, you indicated that [The data produced and analyzed in the present study are included in the paper and are also available in the supporting Information. Moreover, the corresponding author can supply additional details upon request.].

**Action taken:** We have ensured that all data underlying our study's findings are freely available. The data are included in the manuscript and supporting information files.

This policy applies to all data except where public deposition would breach compliance with the protocol approved by your research ethics board. If your data cannot be made publicly available for ethical or legal reasons (e.g., public availability would compromise patient privacy), please explain your reasons on resubmission and your exemption request will be escalated for approval.

3. Please review your reference list to ensure that it is complete and correct. If you have cited papers that have been retracted, please include the rationale for doing so in the manuscript text, or remove these references and replace them with relevant current references. Any changes to the reference list should be mentioned in the rebuttal letter that accompanies your revised manuscript. If you need to cite a retracted article, indicate the article’s retracted status in the References list and also include a citation and full reference for the retraction notice.

**Action taken:** We have reviewed our reference list to ensure it is complete and accurate

**Reviewer #1 Comments and Responses:**

1. **Comment:** *The abstract is better presented as a single paragraph.*
   - **Response:** We have revised the abstract to present it as a cohesive paragraph. Additionally, we have included a statement about the study's significance in relation to current HBV treatment options to provide a more comprehensive overview.
2. **Comment:** *Authors should elaborate more on validating the pharmacophoric model through using more than one validation approach; cost analysis, decoy set, and/or Fischer's method.*
   - **Response:** We have revised the method section in the revised manuscript. Accordingly, the Drugbank was used as a decoy to validate the PharmIt screening and ensure that the model was validated to yield flavonols potentially. The wording has been revised.
3. **Comment:** *Chemical structures or even the SMILES strings of the identified hits should be presented. Additionally, aligning the identified hits with the deduced pharmacophoric model should be presented.*
   - **Response:** We have incorporated the identified hits' chemical structures and SMILES strings into the results section in a new Result section and a new Figure. Furthermore, we have updated the S2 Table by including the STRINGS and predicted biological activity based on the QSAR model for the screened flavonols.
4. **Comment:** *Concerning the discussion/conclusion, authors are advised to elaborate more on the future of this work. What are the study limitations and what approaches could be conducted to further address them?*
   - **Response:** The discussion and conclusion sections have been expanded to address the future implications of this work, study limitations, and potential future approaches. We discuss the limitations, such as the limited diversity of flavonoids examined and possible biases in the screening process, and propose strategies for further research.

**Reviewer #2 Comments and Responses:**

1. **Comment:** *Title: It could be more concise e.g., "Pharmacophore Modeling and QSAR Analysis of Anti-HBV Flavonols."*
   - **Response:** We have revised the title to "Pharmacophore Modeling and QSAR Analysis of Anti-HBV Flavonols" to make it more concise and reflective of the study's content.
2. **Comment:** *Abstract: Include a brief statement about the study's significance in relation to current HBV treatment options.*
   - **Response:** A statement about the study's significance in the context of current HBV treatments has been added to the abstract to highlight the relevance and potential impact of the research findings.
3. **Comment:** *Methods: Certain technical terms may require clarification for readers who are not well-versed in the field. For example, a brief explanation of "Euclidean distance calculations" would be helpful.*
   - **Response:** We have revised the methods section to briefly explain technical terms such as "Euclidean distance calculations" to ensure clarity for readers who may not be familiar with these concepts.
4. **Comment:** *Results: Some results lack adequate context or explanation. For example, when discussing the ROC curve, it would be beneficial to clarify what the specific AUC values indicate about the model's effectiveness.*
   - **Response:** Additional context and explanation have been added to the results section, particularly regarding the ROC curve and AUC values, to clarify the model's effectiveness in identifying potential anti-HBV flavonols.
5. **Comment:** *Discussion: While the strengths of the models are highlighted, it is also important to address potential limitations and areas for improvement. I suggest that a paragraph discussing the study's limitations, such as possible biases in the screening process or the limited diversity of the flavonoids examined, should be added.*
   - **Response:** We have added a paragraph in the discussion section addressing the study's limitations, including potential biases in the screening process and the limited chemical diversity of flavonoids examined. We also discuss areas for improvement and future research directions.
6. **Comment:** *General Comments: There are grammatical errors and awkward phrasing throughout the text, and a thorough proofreading is therefore highly recommended to identify any remaining errors and improve overall clarity.*
   - **Response:** We have thoroughly proofread the manuscript, correcting grammatical errors and awkward phrasing to improve overall clarity and readability.

We believe these revisions have addressed the reviewers' comments and significantly improved the manuscript. We hope that the revised version meets the standards of PLOS ONE and look forward to your favorable consideration.

Thank you for your time and the opportunity to revise our manuscript.

Sincerely,

Alireza Mohebbi
Department of Virology, School of Medicine, Iran University of Medical Sciences, Tehran, Iran
Tel: +98 935 467 4593; Email: [Alirezaa2s@gmail.com](mailto:Alirezaa2s@gmail.com)
